# Celastrol suppresses neovascularization in rat aortic vascular endothelial cells stimulated by inflammatory tenocytes via modulating the NLRP3 pathway

**DOI:** 10.1515/med-2024-1121

**Published:** 2025-05-06

**Authors:** Yong Yang, Huajun Wang, Huige Hou, Jiwen Chen, Xiaolei Chen, Hongjian Zheng, Kai Zheng, Baofei Ye, Chunhui Wu, Xiaofei Zheng, Shiguo Yuan, Boyuan Zheng

**Affiliations:** Department of Orthopedics, The Affiliated Shunde Hospital of Jinan University, Foshan, Guangdong Province, China; Department of Sports Medicine, The First Affiliated Hospital, Guangdong Provincial Key Laboratory of Speed Capability, The Guangzhou Key Laboratory of Precision Orthopedics and Regenerative Medicine, Jinan University, 510630, Guangzhou, China; Department of Orthopaedic, Hainan Hospital, Guangdong Provincial Hospital of Chinese Medicine, Guangzhou University of Chinese Medicine, Guangzhou, China; Department of Orthopaedic, Hainan Traditional Chinese Medicine Hospital, Hainan Medical University, Hainan, China

**Keywords:** celastrol, neovascularization, angiogenesis, inflammation, tenocytes, NLRP3, rotator cuff tear

## Abstract

Appropriate formation of blood vessels is critical for tendon-bone healing during tendon injury, as excessive angiogenesis would exacerbate scar formation and lead to chronic pain and dysfunction. The mechanism to regulate inflammatory angiogenesis during tendon-bone healing remains to be elucidated. Here, we utilized lipopolysaccharide (LPS) to induce tenocyte inflammation and applied the conditioned medium from inflammatory tenocytes to treat rat aortic vascular endothelial cells (RAOECs). The results that indicated LPS treatment significantly induced the mRNA and protein upregulation of NLRP3, tumor necrosis factor α, IL-1β, and vascular endothelial growth factor A (VEGFA), as well as the secretion of VEGFA. Furthermore, the conditioned medium stimulated RAOEC angiogenesis. Celastrol, a quinone-methylated triterpenoid from *Tripterygium wilfordii*, has been reported to treat osteoarthritis. Here, celastrol could suppress LPS-induced upregulation of NLRP3 and IL-1β, and the secretion of VEGFA. Celastrol treatment also suppressed the conditioned medium-induced angiogenesis in RAOEC. Moreover, we established a rotator cuff tear rat model to stimulate tendon injury. Celastrol administration at the lesion significantly promoted tendon healing and functional recovery in injured mice by regulating NLRP3 and VEGFA levels. Taken together, these data suggest that the inflammation-induced tenocyte injury leads to angiogenesis, and that celastrol administration could suppress the inflammatory tenocyte-induced angiogenesis to promote tendon-bone healing via the NLRP3 pathway.

## 1 Introduction

Shoulder rotator cuff injury is a common shoulder injury that leads to recurring joint pain and severe mobility impairment, with the incidence accounting for 50–85% of shoulder joint diseases [1,2]. It is reported that approximately 40% of shoulder pain is due to rotator cuff injuries, and every year there are 4.5 million patients seeking treatment, with nearly 250,000 cases needing surgery [3,4]. Like other sports-related injuries, rotator cuff injuries impose a significant economic and psychological burden on society [5,6]. Currently, there are various clinical approaches to treat rotator cuff injuries, mainly categorized as surgical and non-surgical treatments. Non-surgical treatments typically include biophysical stimulation, rehabilitation training, steroid hormone, or non-steroidal anti-inflammatory drug injections [7,8]. Surgical treatment is the primary modality for clinical management of rotator cuff injury. Despite the increasing maturity of surgical repair techniques, the postoperative re-tear rate remains as high as 25–75% [9,10].

The mechanism of tendon-bone healing is a complex process that involves a series of factors, including stem cells, biological factors, immune-inflammatory responses, and vascular regeneration [11]. Increasing evidence indicates an inseparable relationship between inflammation and tendinopathy [12]. Recent studies have shown elevated levels of various pro-inflammatory mediators, such as PGE2, IL-1, and IL-6 [13]. When compared to normal tendon tissue, early-stage tendon tissues exhibit activation of downstream signaling pathways, such as IFN, NF-κB, and STAT-6 and that of late-stage shows the activation of M2 microglia and STAT-6 pathway [14]. Inflammation response also contributes to the angiogenesis [15]. Uncontrolled angiogenesis is closely related to many diseases, such as tendinitis, arthritis, psoriasis, and cancer [16]. Unregulated proangiogenic factors induced by inflammation may exacerbate scar formation and improper tissue healing would [17] be delayed by large amounts of extracellular matrix [18]. High proangiogenic pressure may lead to the dysfunction of early vasculature and controlling blood vessel density would increase the functionality of early vasculature and long-term outcomes [19]. VEGF is one of the important factors that regulate tissue vascular generation [20,21]. It is mainly expressed in endothelial cells but also expressed in various non-endothelial cells [22]. Its function is generally considered to be related to vascular generation in local tissues. Inflammatory mediators like tumor necrosis factor-α (TNF-α) and IL-6 can up-regulate vascular endothelial growth factor A (VEGFA) and other angiogenic factors, thereby linking inflammation to angiogenesis [23]. Thus, to elucidate the mechanism under inflammation induced angiogenesis needs to be explored in detail and further control of angiogenesis would reduce vascular regression, edema, and promote tendon-bone healing.

Celastrol is a quinone triterpenoid compound extracted from the roots of the plant *Tripterygium wilfordii* [24]. Its molecular formula is C_29_H_38_O_4_, with very low toxicity it has gained widespread attention for its immunosuppressive and anti-inflammatory properties [25,26], against many diseases, such as rheumatoid arthritis [27,28], systemic lupus erythematosus [29], allergic skin inflammation [30], inflammatory bowel disease [31], and cancers [32]. The anti-inflammatory effects of celastrol can be summarized as follows: regulating the production of chemotactic factors [32]; modulating the function of inflammatory cells, including macrophages [33]; regulating the expression of inflammatory mediators [26]; and regulating osteoclast differentiation [34]. However, the role of celastrol in regulating rotator cuff injures remains to be supplemented.

Here, in the current study, we developed a model of lipopolysaccharide (LPS)-induced tenocyte inflammatory response and tenocytes-induced rat aortic vascular endothelial cell (RAOEC) angiogenesis. We tried to elucidate the role of celastrol on these processes *in vitro* and the mechanism in promoting tendon-bone healing in a rotator cuff tear (RCT) model *in vivo*.

## 2 Materials and methods

### 2.1 Animal use

Sprague-Dawley (SD) rats weighting about 100 g (5–6-week-old, female) were purchased from the Guangdong medical laboratory animal center.

### 2.2 Cell culture

Primary rat tenocytes were isolated from SD rats. Briefly, for tendon culture, the cervical dislocation method was applied, and the tendons of the extremities were isolated and cut into pieces. Then the cut tissues were attached to the bottom of a culture bottle and cultured with DMEM (Gibco, CA, USA) with 10% FBS (Sigma-Aldrich, MO, USA) and 1% antibiotic-antimycotic (Gibco). After 6 h, the tissue blocks were removed and washed with PBS, and digested with 0.25% trypsin (Gibco), and passed down for 3 or 4 passages before usage. Tenocytes were seeded in six-well plates with the concentration of 1 × 10^5^/mL and cultured for 1 day before the administration of LPS (0.5 or 1 μg/mL, Sigma, USA). The RAOEC line was purchased from Yaji Biological Co., Ltd (#YS2272C, Shanghai, China).

### 2.3 Tube formation assay

To assess the angiogenic potential of substances secreted by tenocytes after exposure to LPS, a modified tube formation assay was utilized [35]. These media collected from LPS-exposed tenocytes were used to coat 96-well plates with a Matrigel layer (BD, USA). RAOECs were then suspended in either the LPS-stimulated tenocyte media or the control medium for a duration of 24 h. Subsequently, about 10,000 cells were dispensed into each well for a 6 h incubation period to form the capillary-like structures. The images were captured using a digital imaging system (Axio Observer, Zeiss, Germany) and then were quantitatively evaluated using AngioTool (NIH, USA) [36].

### 2.4 ELISA

Tenocytes were treated with or without LPS, or with other treatments with or without celastrol administration. Then the supernatants were collected and rat VEGFA secreted proteins from supernatants were determined by ELISA analysis using VEGFA (#PV960, Beyotime, Shanghai, China) ELISA kits, and the concentration was measured by a Bio-Rad enzyme-linked immunosorbent assay reader (Bio-Rad, USA).

### 2.5 Rat RCT model

Rat RCT model construction was performed as we previously reported [37]. Briefly, a 1.5 cm cut was made along the front and outer aspect of the shoulder. This allowed for the exposure of the supraspinatus muscle and its tendon. Next, a precise division of half of these tendons from the greater tuberosity was performed. Following this, the tendons of the supraspinatus were carefully stitched in successive layers with a suture size of 4-0. Then rats were randomly divided into three groups: Sham, RCT, RCT + Celastrol groups. RCT + Celastrol group, rats were injected with 1 mg/kg celastrol (Sigma-Aldrich, Darmstadt, Germany) intra-articularly per week. Four or eight weeks later, tendons were subjected to biomechanical testing.

### 2.6 Biomechanical testing

Biomechanical testing was also performed as we previously reported [37]. Briefly, at intervals of 4 or 8 weeks post-RCT construction, the full-length tendons from the rat’s supraspinatus muscle along with the proximal part of the humerus were harvested for biomechanical evaluation. This evaluation was conducted using an MTS 858 material testing system (MTS System, Minneapolis, MN, USA), initiating the procedure with a preload range up to 5 N, to a constant extension rate of 14 mm/s. The maximal load the tendons could withstand before failure (measured in Newtons) and their stiffness (measured in Newtons per millimeter) were both determined and logged using a Sigma-Aldrich Plot 8.0 software application (Sigma). The ultimate force was ascertained using the load versus displacement graph, whereas stiffness was derived by calculating the gradient of the slope.

### 2.7 RNA isolation and real-time qPCR

Total RNA was isolated utilizing the TRIzol reagent (Invitrogen, Carlsbad, CA, USA), adhering to the protocol provided by the supplier. The conversion of mRNA to cDNA was carried out using the EasyScript cDNA Synthesis SuperMix (TransGen Biotech, Beijing, China). For the synthesis of cDNA, the TransScript mRNA First-Strand cDNA Synthesis SuperMix (TransGen) was employed. Quantitative real-time PCR analyses were conducted with the aid of the TransStart Top Green qPCR SuperMix (TransGen). The specific primer sequences that were used can be found in Table 1.

**Table 1:** Primer sequences for real-time qPCR

Gene	Forward (5′–3′)	Reverse (5′–3′)
*IL-1β*	CCAGGACATGCTAGGGAGC	CAGAGGCAGGGAGGGAAA
*Tnf-α*	AAAACTCGAGTGACAAGCCCGT	TCCCTTGAAGAGAACCTGGGAG
*Nlrp3*	CAGAAGCTGGGGTTGGTGAA	CCCATGTCTCCAAGGGCATT
*Vegfa*	GCACATAGGAGAGATGAGCTTCC	CACGCCTTGGCTTGTCACAT
*Gapdh*	TGATTCTACCCACGGCAAGTT	TGATGGGTTTCCCATTGATGA

### 2.8 Western blotting

Using RIPA buffer supplemented with protease inhibitors from Beyotime, China, the samples were lysed. The proteins were then resolved using 10% SDS-PAGE and subsequently transferred onto PVDF membranes sourced from Millipore. After blocking with milk, the membranes were treated with primary antibodies specific to NLRP3 (#ab263899; Abcam, Cambridge, UK), IL-1β (#EPR21086; Abcam), and GAPDH (#ab8245; Abcam). This was followed by a 1 h room temperature incubation with secondary antibodies from Abclonal Biotechnology, Wuhan, China. The blots were visualized utilizing an enhanced chemiluminescence method provided by Beyotime. For normalization purposes, GAPDH serves as the internal standard.

### 2.9 Immunocytochemistry

Tenocytes were grown on coverslips coated with poly-l-lysine in 24-well plates. After treatment, the cells were fixed using a 4% paraformaldehyde solution in PBS. For immunolabeling, the cells were incubated at 4°C overnight with the following primary antibody: NLRP3 (#ab263899; Abcam, Cambridge, UK), and post-primary antibody incubation, the cells were washed and then incubated with the appropriate AlexaFluor 488 conjugated secondary antibodies (Invitrogen). After further washing with PBS, the cells were mounted using DAPI-Fluoromount. The samples were then imaged with a LSM 880 confocal microscope (Carl Zeiss, Germany).

### 2.10 Statistical analyses

All experiments were conducted from three repeated experiments and subjected to statistical analysis using SPSS software version 27.0 to calculate the mean value and standard deviation. Student’s *t*-test was employed for the analysis of two independent samples. In cases where comparisons involved three or more groups, a one-way ANOVA was utilized. All the experiments were repeated at least three times. A *P*-value of less than 0.05 was considered to indicate statistical significance.


**Ethical approval:** All animal procedures were approved by the Ethics Committee of the First Affiliated Hospital, Jinan University (202108246-25), and were conducted in accordance with institutional guidelines for animal care and use.

## 3 Results

### 3.1 Conditioned medium from LPS-induced tenocytes induces RAOEC angiogenesis

**Figure 1:**
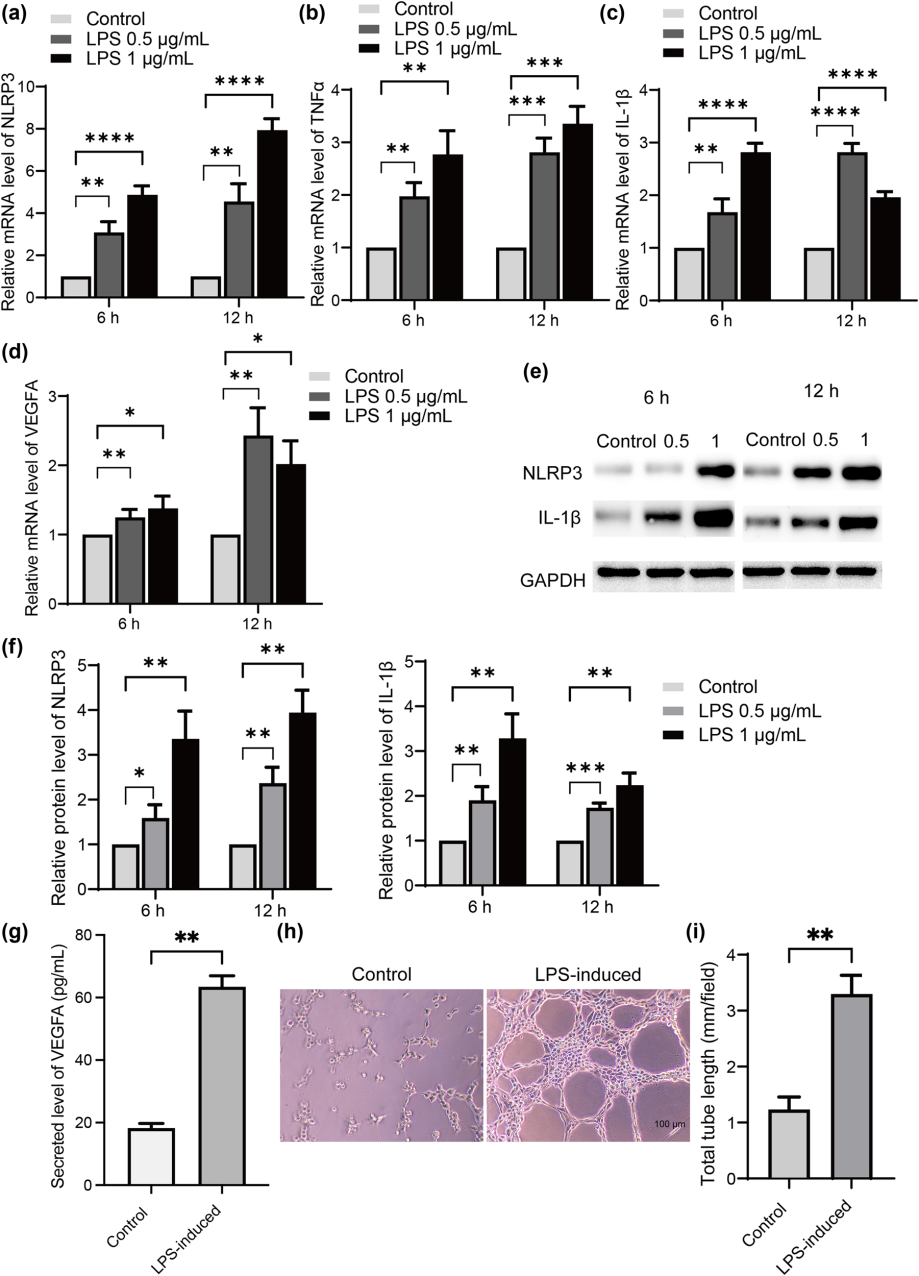
Conditioned medium from LPS-induced tenocytes induces angiogenesis. (a) Cultured tenocytes were treated with LPS at different concentrations for 6 or 12 h. Then the mRNA levels of NLRP3 (a), TNF-alpha (b), IL-1beta (c), and VEGFA (d) were determined. The protein levels of NLRP3 and IL-1beta were also detected (e) and (f) by western blotting. (g) The secreted levels from LPS-treated tenocytes were determined by ELISA. (h) RAOEC cells were treated with conditioned medium from LPS-induced tenocytes and then subjected to a tube formation assay. The representative images are shown. Scale bar, 100 μm. The total tube length was quantified and shown (i). Data are shown as mean ± SD from three repeated experiments (*n* = 3). *denotes *P* < 0.05; **denotes *P* < 0.01; ***denotes *P* < 0.001; ****denotes *P* < 0.0001.

To determine the role of tenocyte inflammation during angiogenesis, the primarily cultured tenocyte was treated with different concentrations of LPS (0.5 or 1 μg/mL for 6 or 12 h). As shown in Figure 1, LPS administration significantly induced the mRNA expressions of NLRP3, TNFα, and IL-1β in tenocytes, in a time- and concentration-dependent manner (Figure 1a–c). The results showed that LPS also significantly induced the mRNA upregulation of VEGFA in tenocytes (Figure 1d). Protein levels of NLRP3 and IL-1β were significantly upregulated upon the treatment of LPS (Figure 1e and f), with the highest levels observed at a concentration of 0.5 μg/mL for 12 h. Thus, this condition was chosen for the subsequent cell experiments. Moreover, the ELISA assays revealed that LPS markedly induced the secreted level of VEGFA in cultured medium of tenocytes (Figure 1g). Then cultured RAOEC cells were treated with this conditioned medium for tube formation assay. The data showed that medium from LPS-treated tenocyte significantly induced the angiogenesis in RAOECs. Taken together, these results showed that LPS-induced inflammation in tenocytes and the conditioned medium from inflammatory tenocytes induced angiogenesis of RAOECs.

### 3.2 Celastrol suppresses LPS-induced inflammation in tenocytes via NLRP3

**Figure 2:**
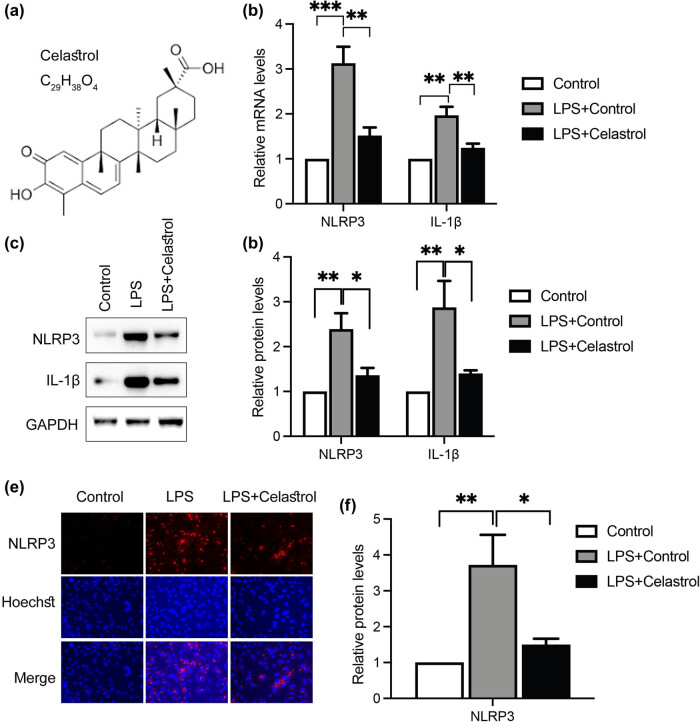
Celastrol suppresses LPS-induced inflammation in tenocytes via NLRP3. (a) Chemical formula and structure of celastrol. Tenocytes treated with LPS, with or without celastrol, were subjected to RT-qPCR or western blotting assays to determine the mRNA levels of NLRP3 and IL-1β (b), and the protein levels of NLRP3 and IL-1β (c) and (d). Tenocytes were also subjected to immunocytochemistry for the detection of NLRP3 expression (e) and (f). Scale bar, 10 μm. Data are shown as mean ± SD from three repeated experiments (*n* = 3). *denotes *P* < 0.05; **denotes *P* < 0.01; ***denotes *P* < 0.001.

Celastrol (the structure is shown in Figure 2a) has been reported to suppress inflammation [38]; however, the role in tenocyte inflammation has not been determined. Thus, the administration of celastrol was applied to LPS-induced tenocytes. As shown in Figure 2b, the upregulated mRNA levels of NLRP3 and IL-1β by LPS treatment were significantly suppressed after the administration of celastrol. The protein levels of NLRP3 and IL-1β were also significantly downregulated by celastrol revealed by western blotting assay (Figure 2c and d). Furthermore, the immunocytochemistry assays also showed that celastrol could suppress the expression of NLRP3 in tenocytes (Figure 2e and f). Taken together, these data suggest that celastrol suppresses LPS-induced inflammation in tenocytes via regulation of NLRP3.

### 3.3 Celastrol suppresses angiogenesis induced by inflammatory tenocytes

**Figure 3:**
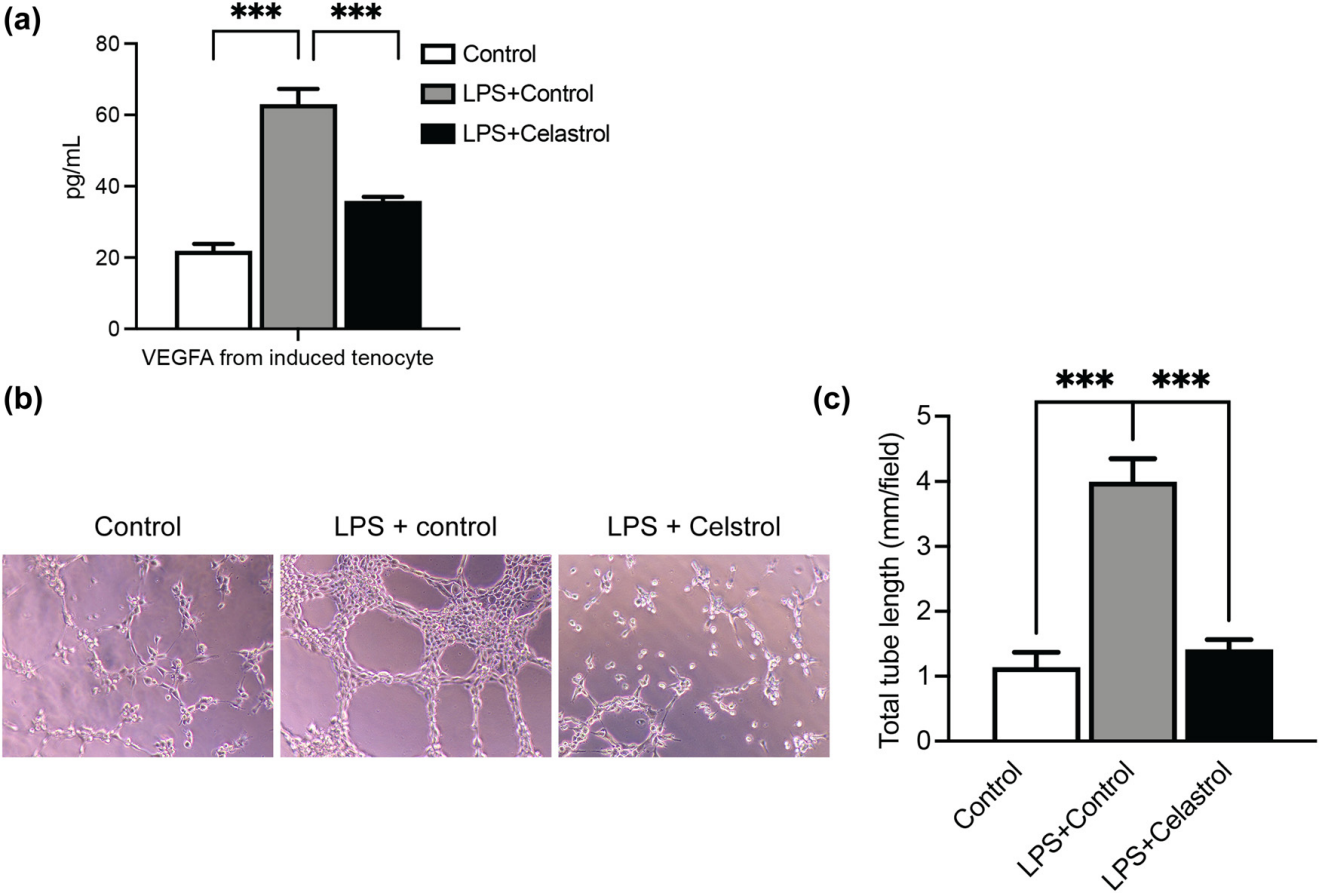
Celastrol suppresses angiogenesis by inflammatory tenocytes. (a) LPS-induced tenocytes were treated with or without celastrol, and then the secreted levels of VEGFA from the culture medium were determined. (b) and (c) RAOEC cells were administered conditioned medium from tenocytes with the indicated treatments. Then the angiogenesis assay was conducted. Scale bar, 100 μm. Data are shown as mean ± SD from three repeated experiments (*n* = 3). ***denotes *P* < 0.001.

To verify the role of celastrol in the tube formation ability of RAOECs, we determined the effect of celastrol on the secretion of VEGFA level in tenocytes. As shown in Figure 3a, the administration of celastrol significantly suppressed the secretion of VEGFA in LPS-induced tenocytes. Then RAOECs were treated with the conditioned medium from both groups. The results showed that tube-formation ability of RAOECs was significantly inhibited (Figure 3b and c), demonstrating that the celastrol suppresses angiogenesis via regulation of the VEGFA pathway.

### 3.4 Celastrol promotes tendon-bone healing in RCT rats

**Figure 4:**
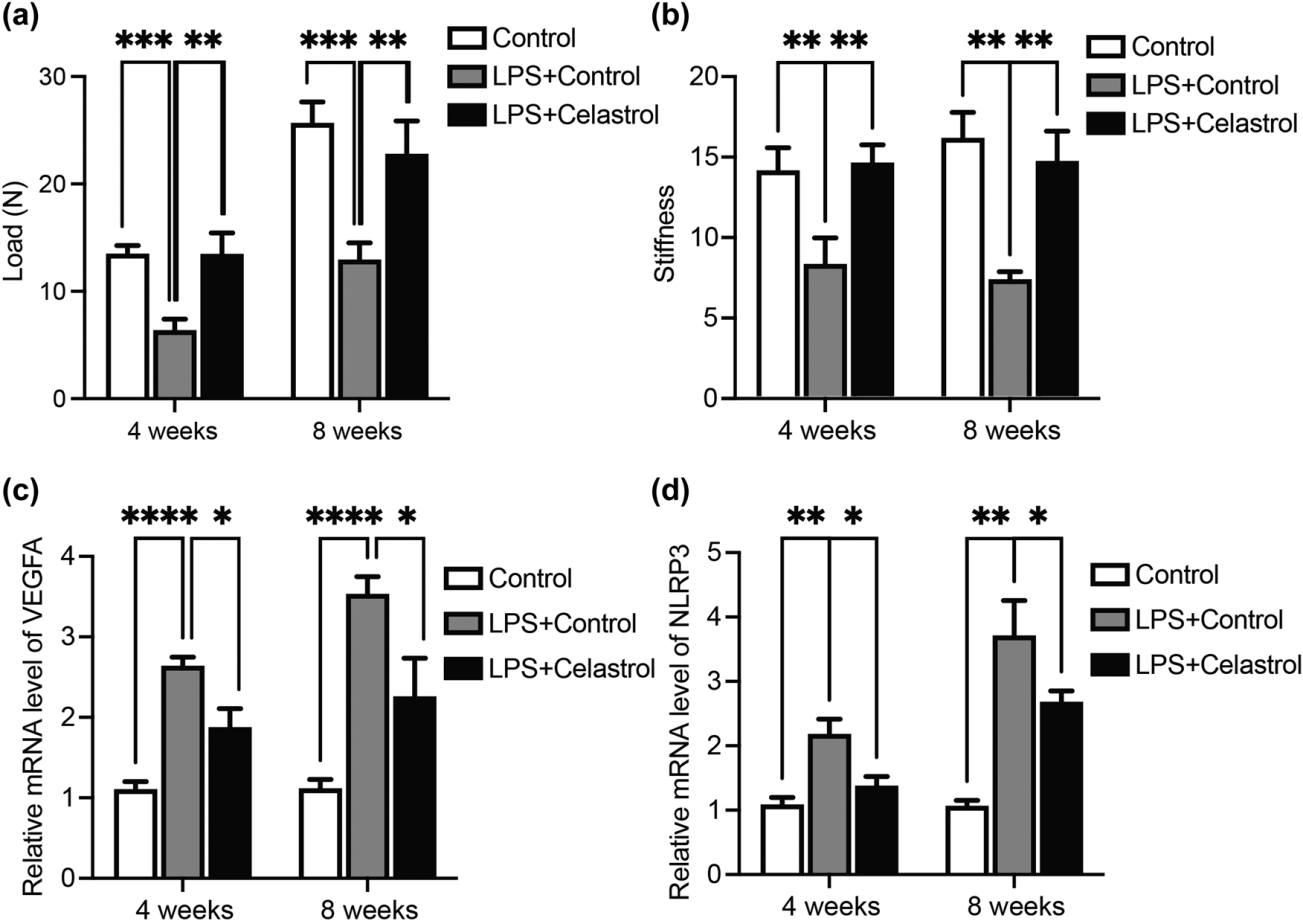
Celastrol promotes tendon-bone healing in RCT. RCT rats were administered LPS with or without celastrol injection. Four or eight weeks later, RCT rats were subjected to mechanical testing. The ultimate load to failure (a) and stiffness test (b) is shown. Then the mRNA levels of VEGFA (c) and NLRP3 (d) from rat tendons were determined. There were three rats in each group at each time points. *denotes *P* < 0.05; **denotes *P* < 0.01; ***denotes *P* < 0.001; ****denotes *P* < 0.0001.

To further elucidate the role of celastrol during tendon injury *in vivo*, we constructed a rat RCT model as we previously reported [37]. After successfully establishing the model, rats were intra-articularly injected with celastrol every week. Then the animals were subjected to biomechanical testing after 4 or 8 weeks, according to a well-established protocol [39,40]. As shown in Figure 4a, the ultimate load to failure was significantly impaired by the injection of LPS; however, this process could be reversed by the administration of celastrol both at 4- or 8-week points. The same trend was observed in stiffness testing, injection of celastrol could significantly rescue the impaired stiffness ability in RCT rats (Figure 4b). Then tendon samples were harvested and subjected to reverse transcription quantitative polymerase chain reaction (RT-qPCR) assay. The data showed that LPS significantly upregulated the mRNA level of both VEGFA and NLRP3 in RCT rats (Figure 4c and d), which could be suppressed by the administration of celastrol. These data indicate that celastrol treatment protects the tenon-bone healing in RCT rats, via modulating VEGFA and NLRP3 pathway.

## 4 Discussion

In this study, we established an inflammatory model in LPS-induced tenocytes. LPS significantly upregulated the inflammatory factors, NLRP3 and IL-1β, and enhanced the secretion of VEGFA. The conditioned medium from LPS-induced tenocytes markedly stimulated the angiogenesis of cultured RAOECs. Furthermore, celastrol, an active component from traditional Chinese medicine, was found to alleviate the levels of NLRP3 and the secretion of VEGFA, hence suppressing the LPS-induced inflammation in tenocytes. Additionally, the administration of celastrol inhibited the angiogenesis of RAOECs triggered by the conditioned medium from LPS-induced tenocytes. Finally, the administration of celastrol significantly improved shoulder function in an RCT animal model. These data point to a novel role of celastrol in the neovascularization of inflammatory tenocytes, achieved by modulating the NLRP3 pathway, both *in vitro* and *in vivo*.

Inflammatory regulation of blood vessel formation during tendon-bone healing is crucial for the recovery of RCT. Due to the limited blood supply and regenerative capacity at the bone-tendon interface, injuries frequently lead to the formation of fibrous scars in the transition zone between bone and tendon, resulting in very slow bone-tendon healing [41]. After RCT, the matrix components and structure become disordered, making it challenging to restore the original biomechanical properties [42]. Angiogenesis regulation is important for the recovery of tendon injury. However, during tissue repair processes, angiogenesis may contribute adverse effects by exacerbating scarring and reducing the post-repair hardness [43]. Factors such as HIF-1α and VEGF would increase carcinogenic signals, leading to poor prognosis due to the upregulation of crucial genes involved in angiogenesis [44]. Restricting rather than promoting blood vessel growth may be more advantageous for the long-term outcome of tissue repair [45].

Deng reported that VEGF can induce cell differentiation, and it has the function of inducing differentiation of bone marrow mesenchymal stem cells into endothelial cells [46]. Sun found that VEGF can promote tendon-to-bone healing by activating Yes-related protein, inducing blood vessel generation and shoulder reconstruction in rats [47]. VEGF not only promotes blood vessel formation in the body but also increases the permeability of blood vessel walls and promotes the growth and proliferation of endothelial cells and perivascular cells, which are essential for self-renewal and regeneration [48,49]. Here, we found that celastrol could not only inhibit the mRNA upregulation but also the secretion of VEGFA in tenocytes, and finally suppressed the blood vessel growth of RAOECs. Our data confirmed that the reduction in angiogenesis would benefit the outcome and promote the recovery of RCT in animal model.

Due to limited metabolic demands and mechanical functions, vascular endothelial growth factors can be completely negligible in healthy tendons [50]. VEGA expression increases in various tendon diseases and tendon healing processes [51]. Following acute tendon injury, vascular endothelial growth factor levels typically increase for a period of time. Boyer et al. detected levels of VEGF in the canine flexor digitorum superficialis tendon at various time points following tendon transection and found that the expression levels of VEGF peaked 1 week later, then gradually decreased, and returned to a lower level in the third week [52]. In contrast to the direct surgical incision tendon injury model mentioned, Petersen et al. [53] and Yoshikawa et al. [54] studied autologous tendon transplantation after anterior cruciate ligament reconstruction and found that the expression of VEGF increased in the late stage after transplantation and then gradually decreased. Riggin et al. [55] demonstrated that local injection of VEGF or anti-VEGF antibody showed that only in the early stage, the vascular response might contribute to tendon healing, but on the contrary, reducing the vascular response may improve healing potential at later time points. In the current study, we found that celastrol suppressed the expression of VEGF, which could inhibit angiogenesis and promote functional recovery in RCT rats. Our results are consistent with the above reports that restricting blood vessel growth is beneficial for tendon-bone healing.

The occurrence of tendon disease is closely related to inflammation. Recent research has shown that in chronic tendon disease, levels of various pro-inflammatory mediators such as PGE2, IL-1, and IL-6 are upregulated compared to normal tendon tissues [56]. In early-stage tendon disease tissues, downstream signaling pathways of M1 and M2 such as IFN and STAT-6 are activated, while in the late stage, the M2 pathway is activated [57]. Clinical studies have shown that treatment with anti-inflammatory drugs such as glucocorticoids during the acute pain period inhibits tendon regeneration and repair, leading to a higher risk of tendon re-rupture, indicating that the inflammatory response in the acute phase promotes tendon repair [58]. Our results also confirmed the upregulated inflammation response during tendon repair.

NLRP3 is one critical component of the NLRP3 inflammasome, which is the most widely studied inflammasome to date [59]. When stimulated in danger, NLRP3 inflammasome could be activated, which promotes the activation of pro-inflammatory cytokines such as IL-1β or other interleukins [60]. NLRP3 protein levels increased in rat RCT tendons [61]. Here, in this study, we found that levels of NLRP3 and IL-1β were upregulated in LPS-induced tenocytes and in rat RCT models, which is consistent with the above studies [62]. Thus, NLRP3 inflammasome is a key regulator during tendon-bone healing. Increased NLRP3 activation may lead to fibrosis, indicating a potential role for NLRP3 in regulating collagen deposition [63]. Blood vessel growth and fibrosis suppress the tendon-bone healing, making easy re-occurrence of RCT.

*T. wilfordii* is found in East Asia, Australasia, Africa, and the Americas and has been used in traditional medicine for years [64]. Celastrol is one of the most abundant bioactive compounds [65]. Research has shown that celastrol possesses various biological activities, such as antioxidant, anti-inflammatory, and anticancer properties [65]. It can be applied in the treatment of many diseases by modulating various molecular targets, including cancer, metabolic diseases, autoimmune diseases, and neurodegenerative diseases [66]. For example, celastrol inhibits the expression of CXCR4 to block the invasion and metastasis of cancer cells [32]. The anti-inflammatory effects of celastrol are mainly attributed to modulating inflammatory signaling pathways, inhibiting the production of inflammatory factors, and regulating the activity of immune cells [67]. Celastrol regulates the anti-inflammatory effect in bone diseases, such as bone healing, osteoarthritis, and osteosarcoma. Deng et al. reported that celastrol could suppress inflammation in rats with advanced arthritis by targeting apoptosis of macrophages and osteoclasts in joints [68]. Celastrol improves osteoarthritis by controlling the TLR2/NF-κB signaling pathway [69]. Celastrol also promotes bone-wound healing in rats of a tibia injury model [70]. Here, consistent with the previous study, we found that administration of celastrol significantly suppressed the inflammation in LPS-induced tenocytes and the subsequent angiogenesis in RAOECs, and inhibited the expression of NLRP3 in RCT rats. Interestingly, celastrol is reported to inhibit the activation of the NLRP3 inflammasome in LPS-induced liver damage and monosodium urate-induced gouty arthritis by blocking the de-ubiquitination of NLRP3 and cleavage of pro-IL-1β, without affecting the total protein level of NLRP3 [71]. We detected the downregulation of cleaved IL-1β, and we did not observe the post-modification but found the downregulation of expression of NLRP3, this may due to the different models induced in different cell types. This controversy should be further clarified in the future study.

There are several limitations in the current study: (1) the detailed molecular mechanism underlying NLRP3/IL-1β signals is lacking, such as pathways related to collagen production [72], ECM disorganization [62], and cytoskeleton dynamics [73]. Particularly, we did not observe other signaling pathways beyond NLRP3/IL-1β underlying celastrol treatment, this could be determined by RNA-seq or proteomic approaches in our future study. (2) We did not determine all the inflammatory response factors, including pro-inflammatory and anti-inflammatory factors, such as IL-2, IL-6, IL-10, etc., also, the secreted compounds more than VEGFA were not included, which would be further supplemented in our future study. (3) While celastrol is highly effective in treating various diseases and can interact with multiple cellular targets, it has limitations, including poor water solubility and low bioavailability. A major concern with the clinical application of celastrol is its narrow therapeutic dose range, which is accompanied by the potential for adverse effects [74]. Additionally, the long-term use of celastrol should be carefully evaluated due to its immunosuppressive effects, which are vital for maintaining normal immune function. Therefore, various strategies should be adopted to mitigate the side effects associated with the clinical application of celastrol.

In conclusion, the present study demonstrates the impact of celastrol on the NLRP3 pathway during LPS-induced tenocyte inflammation, the angiogenesis of RAOEC cells, and functional recovery in RCT rats. These findings suggest that celastrol plays a critical role as a potential anti-angiogenic agent in promoting tendon-to-bone healing during RCT.
